# Supplementary material for: Protein Backbone and Average Particle Dynamics in Reconstituted Discoidal and Spherical HDL Probed by Hydrogen Deuterium Exchange and Elastic Incoherent Neutron Scattering
Source: Biomolecules. 2020 Jan 10;10(1):121. doi: 10.3390/biom10010121 (PMC7022587; doi:10.3390/biom10010121)
Supplement: Supplementary file 1 [file biomolecules-10-00121-s001.pdf]

Supplementary Information

Protein backbone and average particle dynamics in reconstituted discoidal and spherical HDL probed by hydrogen deuterium exchange and elastic incoherent neutron scattering

Valentin Gogonea[1,2,3], Judith Peters[4,5], Gary S. Gerstenecker[1,3], Celalettin Topbas[1,3], Liming Hou[6], Jérôme Combet[4,7], Joseph A. DiDonato[1,2], Jonathan D. Smith[1,2], Kerry-Anne Rye[6], Stanley L. Hazen[1,2,8]

From Departments of [1] Cardiovascular and Metabolic Sciences, Lerner Research Institute[2] Center for Microbiome and Human Health, and [8] Cardiovascular Medicine, Cleveland Clinic, Cleveland, OH 44195; [3] Department of Chemistry, Cleveland State University, Cleveland, OH 44115; [4] Institut Laue Langevin, 6 rue J. Horowitz, BP 156, F-38042 Grenoble Cedex 9, France; [5] Université Joseph Fourier Grenoble I, UFR PhITEM, F-38041 Grenoble Cedex 9, France; [6] Lipid Research Group, School of Medical Sciences, Faculty of Medicine, University of New South Wales, Sydney, Australia 2052; [7] Institut Charles Sadron, CNRS-Université de Strasbourg, 23 rue du Loess, B.P. 84047, 67034 Strasbourg Cedex 2, France.

To whom correspondence may be addressed: Valentin Gogonea, PhD, Department of Chemistry, Science Bld., SI 422, Cleveland State University, Cleveland, OH 44115, Tel.: 216-875-9717; Fax: 216-687-9298; Email: v.gogonea@csuohio.edu

Running title: The dynamics of HDL

Keywords: Apolipoprotein A-I, high density lipoprotein, chemical cross-linking, hydrogen-deuterium exchange mass spectrometry, elastic incoherent neutron scattering, thermal neutron, lipoprotein dynamics, lipoprotein softness, protein backbone dynamics

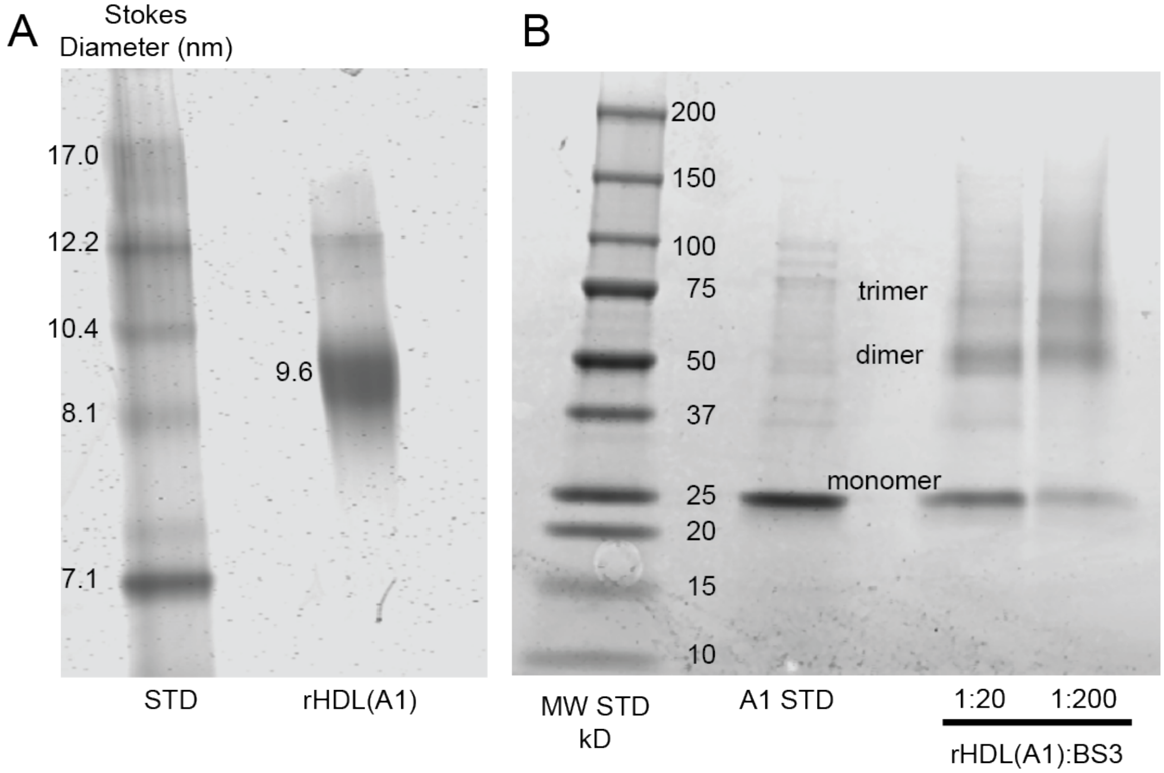

Supplementary Fig. 1. Particle characterization and composition analysis of reconstituted discoidal HDL (rHDL). *A*, Non-denaturing gel of discoidal HDL particles reconstituted with plasma human apoA-I. *B*, SDS page chemical crosslinking gel of rHDL shows that the reconstituted particles contain two apoA-I chains.

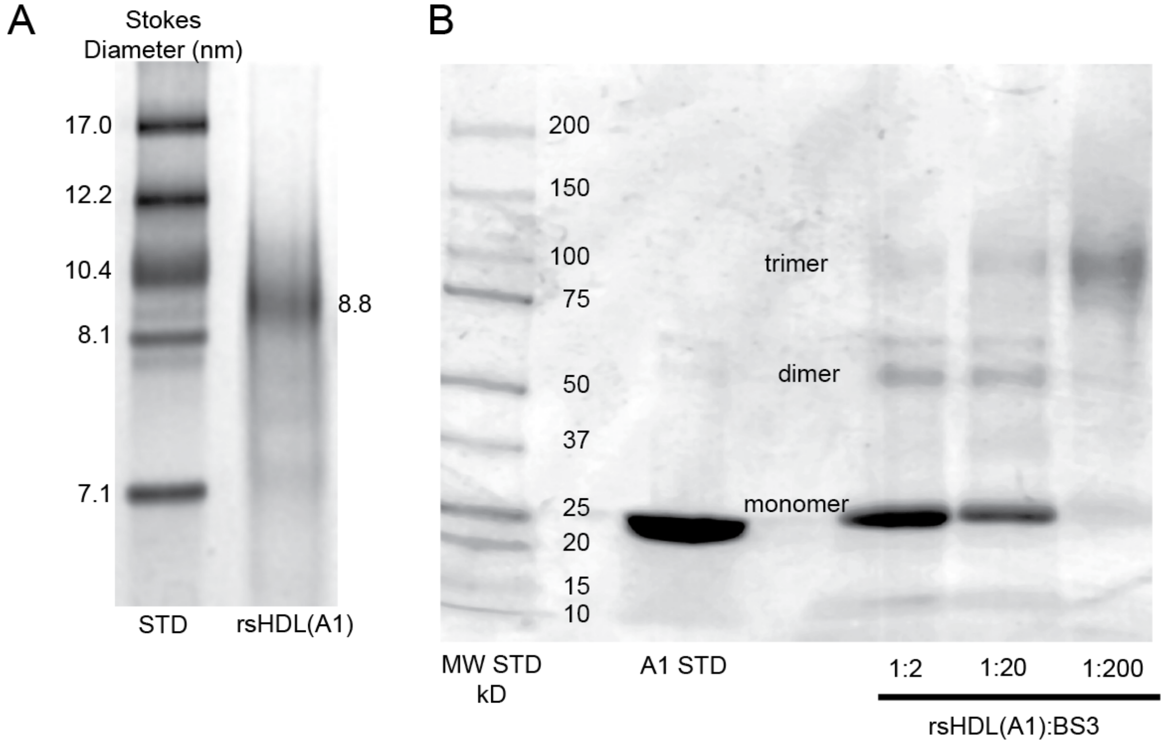

Supplementary Fig. 2. Particle characterization and composition analysis of reconstituted spherical HDL. *A*, Non-denaturing gel of spherical HDL particles reconstituted with plasma human apoA-I (rsHDL). *B*, Crosslinking SDS gel showing that reconstituted spherical HDL preparations contain three apoA-I chains per particle.

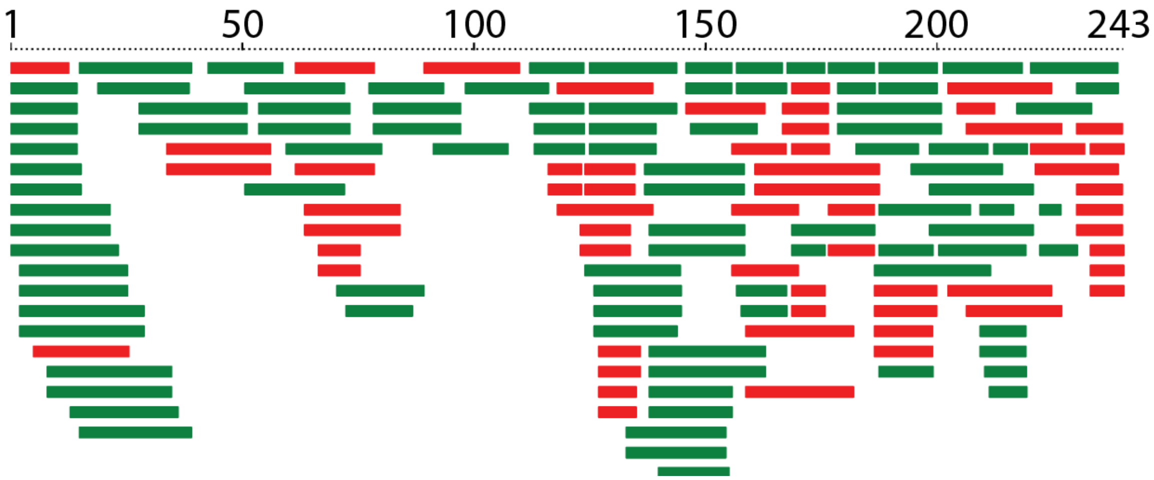

Supplementary Fig. 3. Peptide coverage of full length apoA-I (1-243) in reconstituted sHDL from the HDX-MS experiment. The bar chart shows all overlapping apoA-I peptides obtained by digestion with pepsin (shown as green rectangles) and fungal protease XIII (shown as red rectangles). Pepsin produces many overlapping peptides from the N-terminal of apoA-I, while protease XIII generates many overlapping peptides from the C-terminal of apoA-I in reconstituted spherical HDL.

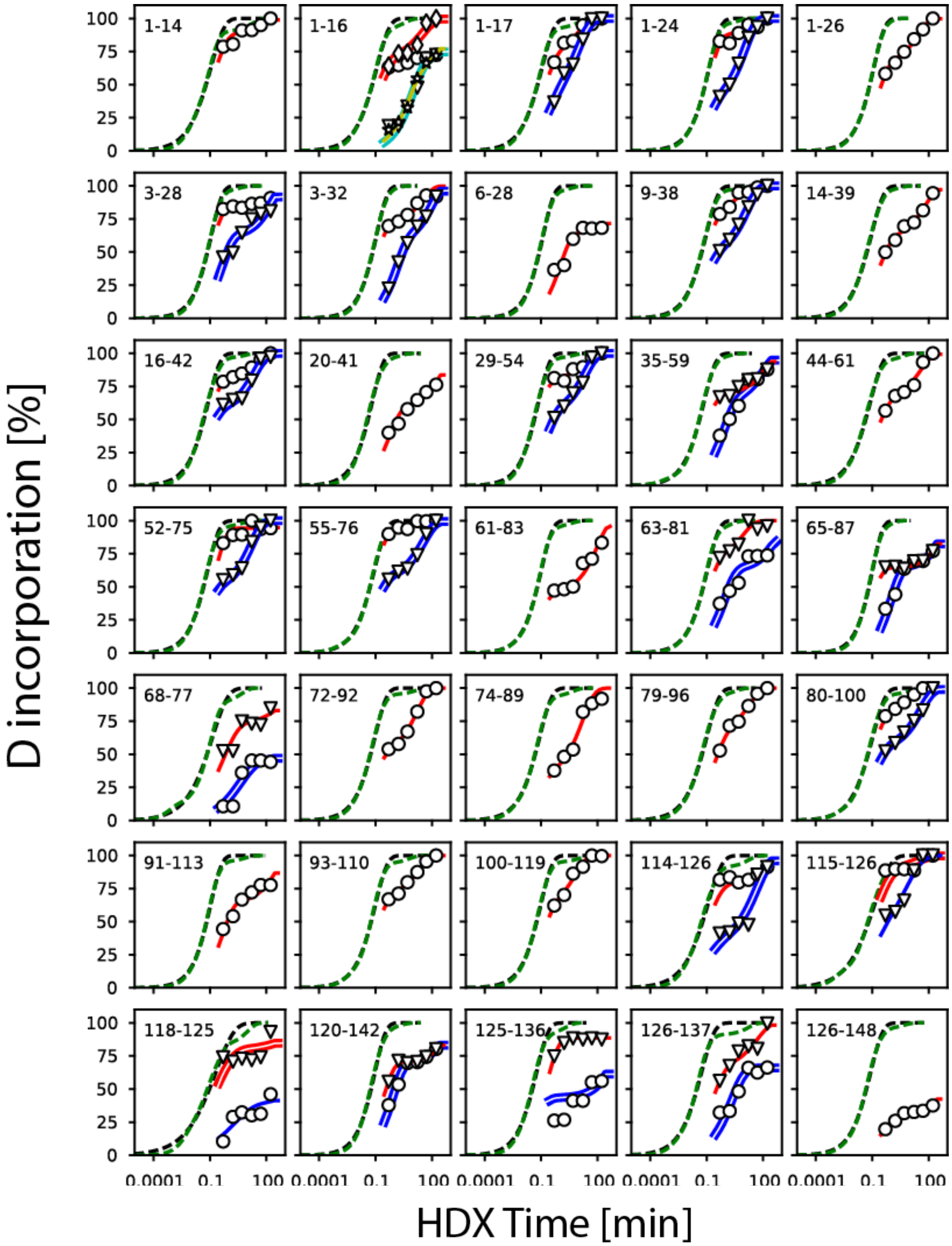

Supplementary Fig. 4. HDX kinetic curves for digested peptides of apoA-I in reconstituted spherical HDL. The green dotted line represents D incorporation values at various HDX times in the predicted random coil peptide. The values were calculated using individual rate constants for residues in the peptide. The black dotted line is the stretched exponential fitted to D incorporation values calculated for the random coil peptide (green dotted line). The D incorporation values measured in the HDX experiment are shown with open circles and triangles for the bimodal HDX kinetics. Stretched exponential curves fitted to the experimental values are shown in red (fast HDX peptide)

and blue (slow HDX peptide). When bimodal HDX is detected the peptide abundance for the slow/fast peptides are usually in a ratio 1:2. The stretched exponential to the experimental data for the peptide with relative intensity 1 is shown as a double line meaning that that peptide originates from two distinct apoA-I chains, while the stretched exponential for the peptide with relative intensity less than 1 is shown as a single line meaning that it originates from a single apoA-I chain. When more than two HDX modes were detected, the data for the third HDX mode are labeled with a diamond sign, etc..

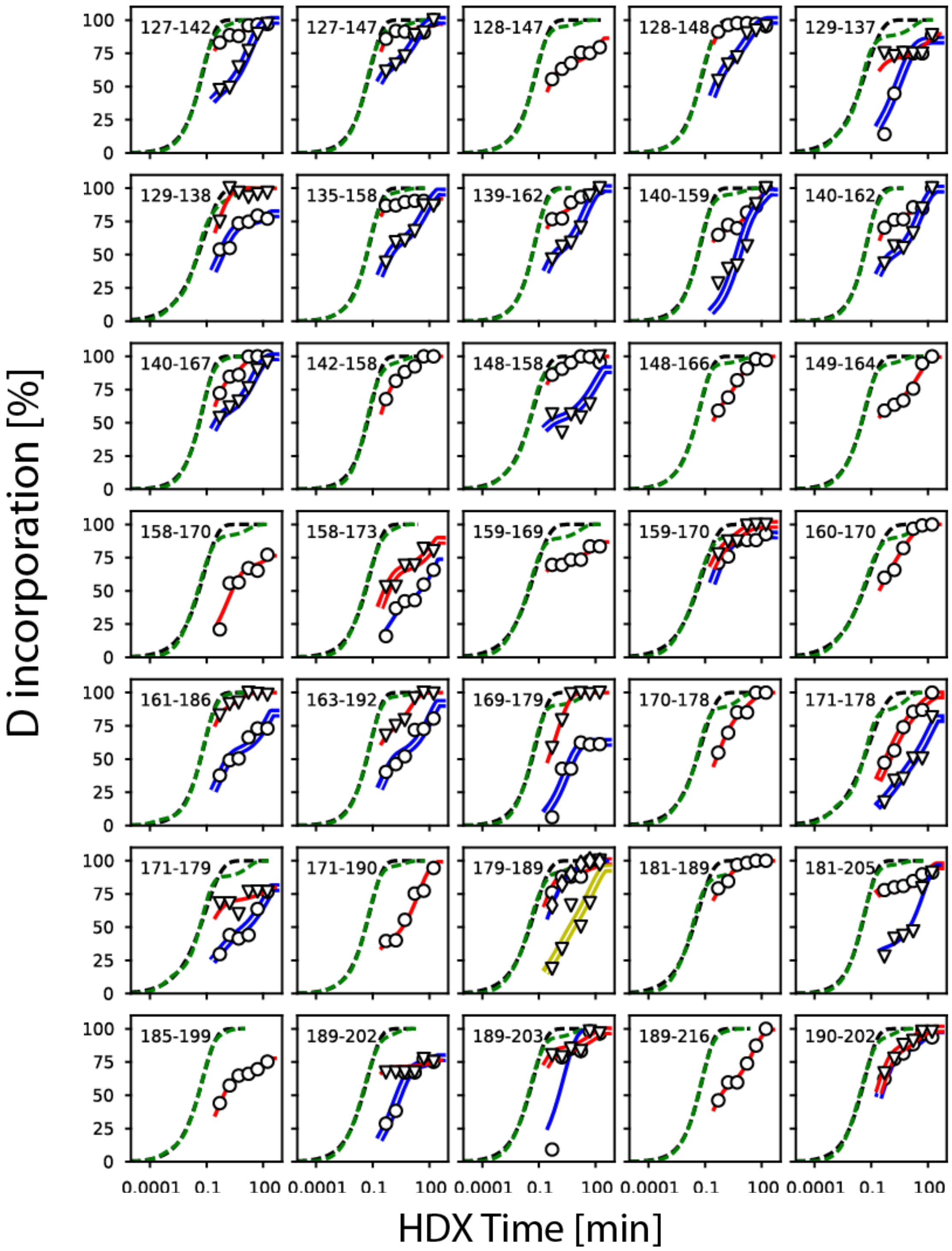

Supplementary Fig. 4. Continued

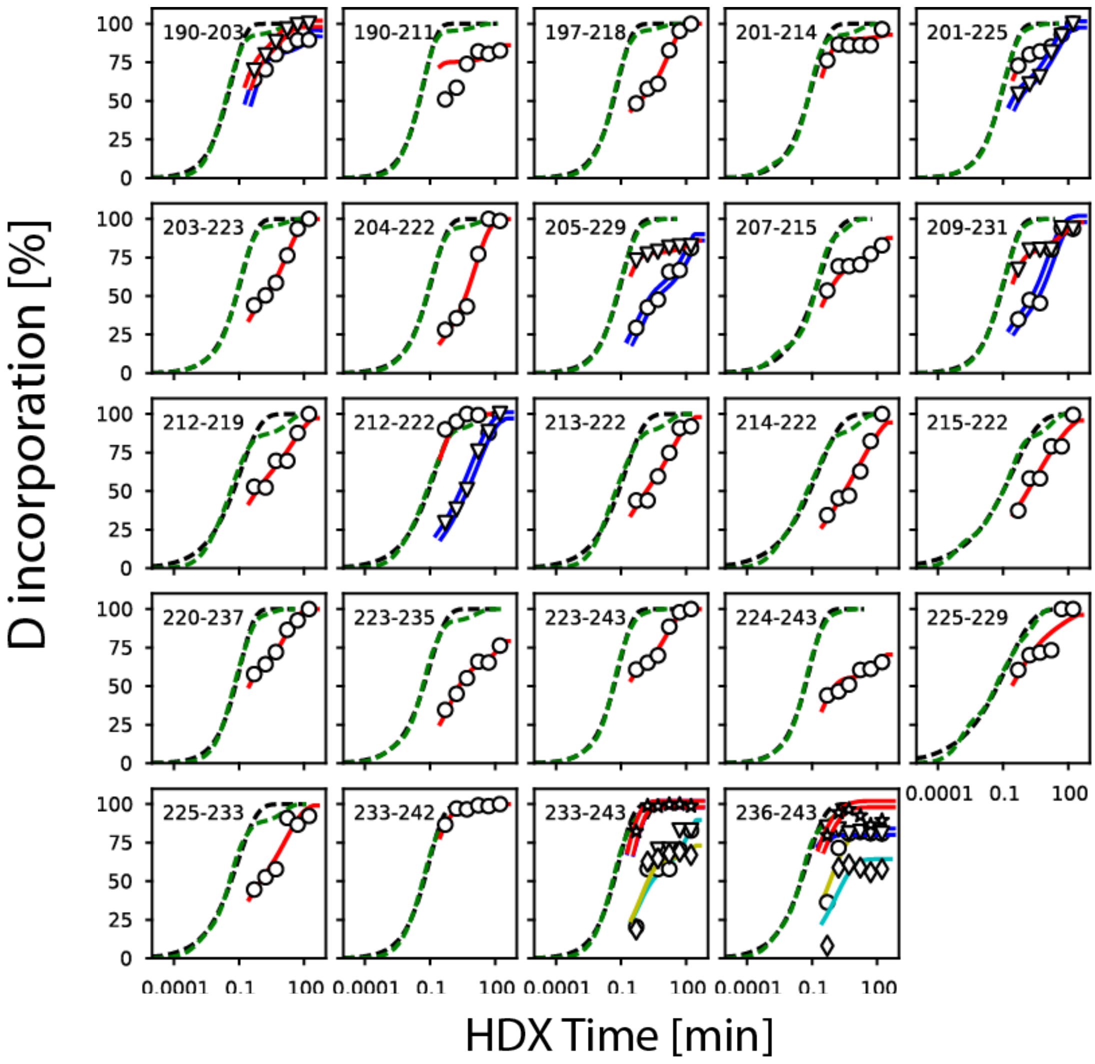

Supplementary Fig. 4. Continued

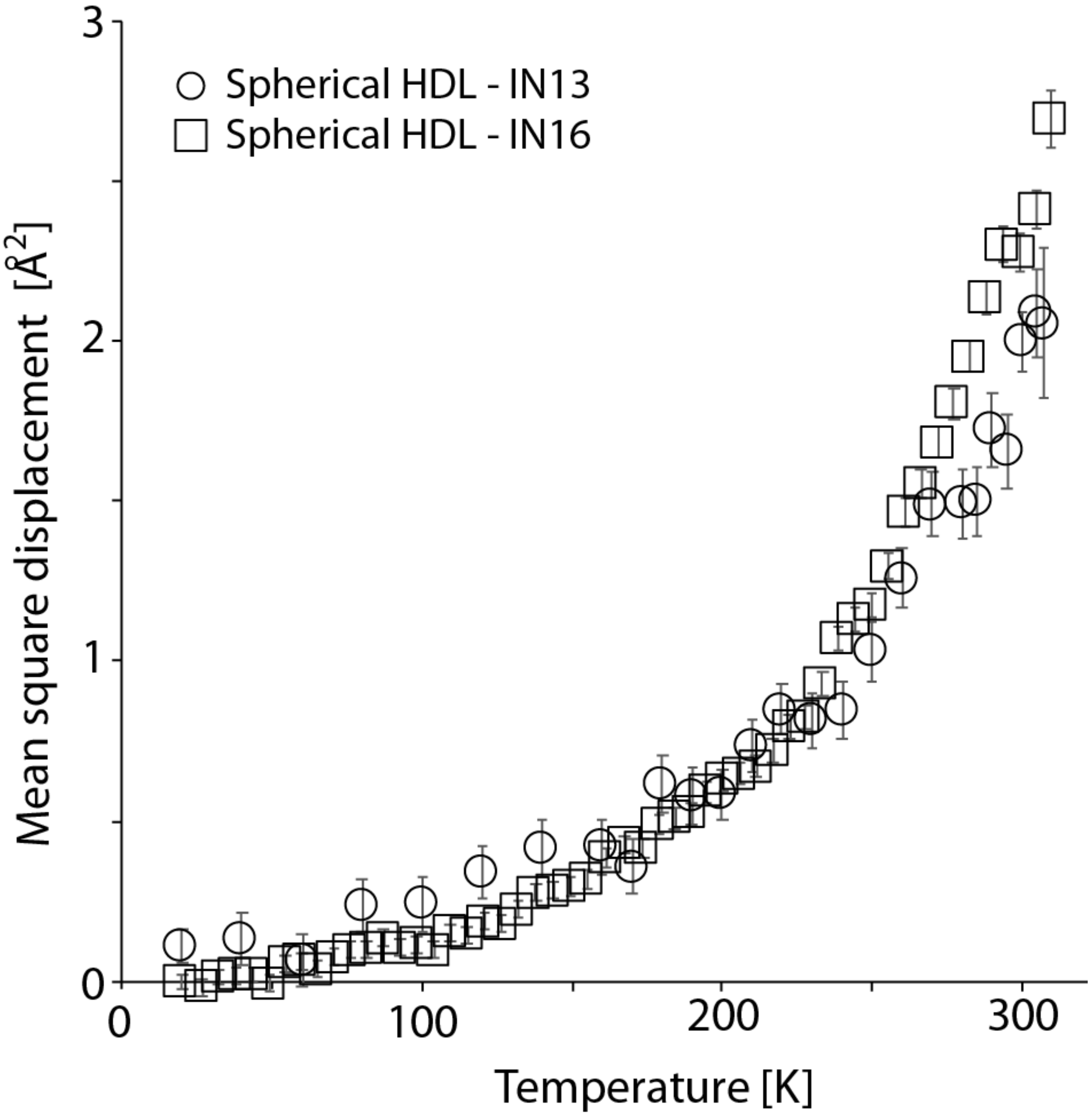

Supplementary Fig. 5. Mean square displacement (MSD) of lipoprotein atoms in reconstituted discoidal HDL and spherical HDL (hydrated powder). The graph shows a comparison of MSD obtained from measurements of sHDL samples on two instruments at Institut Laue-Langevin: IN13 (open circle) and IN16 (open square). The measurement on the IN16 instrument show an increase in lipoprotein dynamics at higher temperatures (> 250 K) within the 1 ns time window compared to the measurement done on the IN13 instrument (time window of 100 ps).

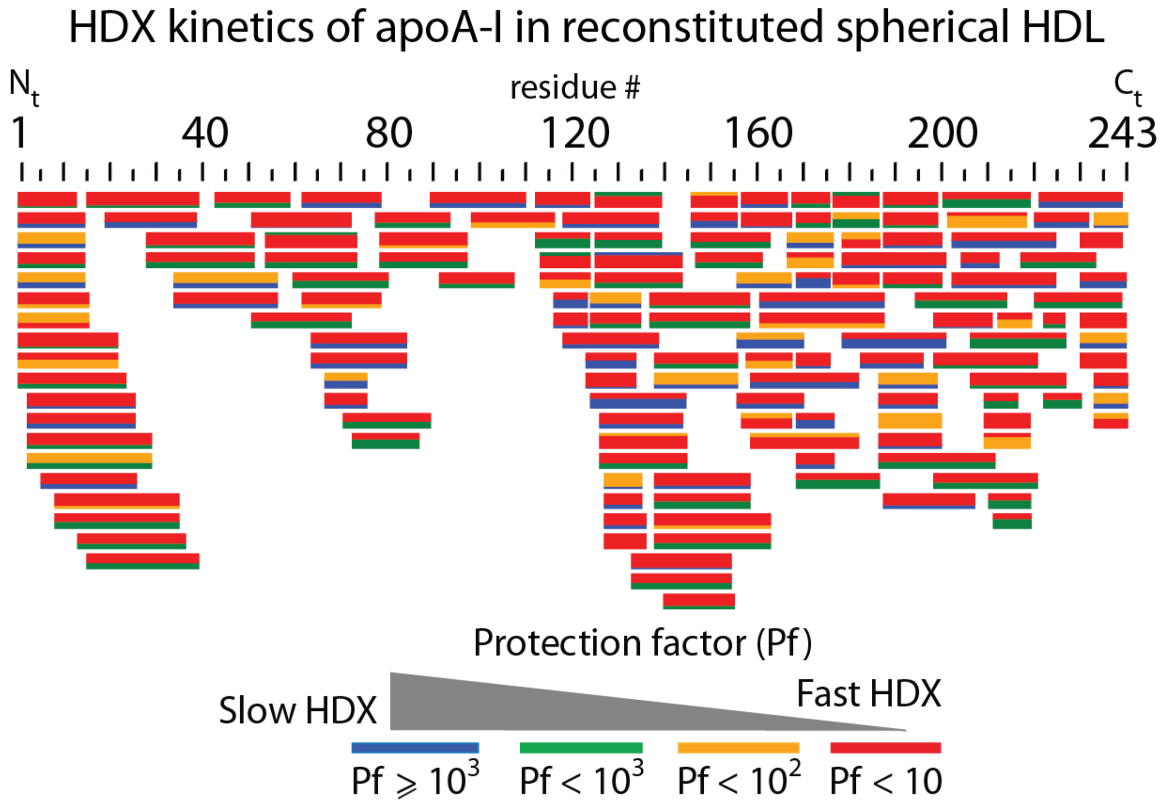

Supplementary Fig. 6. HDX kinetic analysis of apoA-I in reconstituted spherical HDL. The bar chart shows all overlapping apoA-I peptides, resulted from protein digestion, as colored rectangles according to their HDX protection factors. Many of the peptides, which experience biphasic HDX (i.e. having residues exchanging with different HDX rate constant) are displayed by two adjacent stripes with thicknesses proportional with the number of HDX sites with the same HDX rate constant. Bimodal peptides are displayed as individual bars.

Supplementary Table 1. HDX Kinetic Data for Peptides from ApoA-I Chains in the Reconstituted Spherical High Density Lipoprotein obtained at T=0ºC and pH=6.8.

Peptide[a]	k_{rc}[b]	SP[c]	Peptide relative abundance	HDX sites[d]		Pf[e]	
				Phase 1	Phase 2	Phase 1	Phase 2
D_1-L_{14}	8.82	0.61	1	1	7	544	1
D_1-T_{16}[f]	8.38	0.65	0.75	3	6.6	41900	1
			1	3	6.6	25394	84
			0.5	3	6.6	262	1
D_1-V_{17}	8.76	0.66	0.5	3	7.4	796	1
			1	4	6.4	2	842
D_1-D_{24}	7.14	0.72	0.5	2	14	368	1
			1	10	6	77	1
D_1-G_{26}	8.11	0.75	1	6	11.6	221	2
P_3-D_{28}	8.26	0.75	0.5	3	15.4	2772	1
			1	7	11.4	1358	4
P_3-Q_{32}	8.66	0.76	0.5	6	15.6	303	1
			1	9	12.6	802	12
S_6-D_{28}	7.52	0.76	1	6	10.8	15100	8
D_9-L_{38}	10.5	0.69	0.33	5	18.2	56	1
			1	12	11.2	138	1
L_{14}-G_{39}	10.9	0.67	1	8	12	727	3
T_{16}-L_{42}	12.2	0.68	0.5	4	16.8	409	1
			1	9	11.8	278	1
D_{20}-Q_{41}	15.0	0.72	1	7	9.8	4732	1
Y_{29}-T_{54}	13.7	0.73	0.5	4	16	3301	1
			1	9	11	283	2
G_{35}-K_{59}	13.9	0.68	1	7	12.2	1472	1
			0.5	6	13.2	1939	1
L_{44}-R_{61}	12.6	0.64	1	5	8.6	437	2
S_{52}-L_{75}	12.9	0.71	0.5	1	16.6	159851	2
			1	9	8.6	200	1
S_{55}-E_{76}	11.3	0.68	0.75	15	1	1	172

			1	7	9	399	1
R_{61}-R_{83}	9.5	0.73	1	9	7.8	913	1
Q_{63}-G_{81}	9.0	0.7	1	5	8.6	4712	1
			0.5	4	9.6	78	1
T_{68}-K_{77}	7.23	0.53	1	4	3.2	651351	30
			0.5	2	5.2	7749	4
W_{72}-E_{92}	10.7	0.69	1	8	8	166	1
N_{74}-D_{89}	11.9	0.67	1	8	4	191	1
T_{79}-K_{96}	9.8	0.64	1	5	8.6	162	2
E_{80}-Y_{100}	9.31	0.64	0.5	3	12.2	94	1
			1	7	8.2	349	1
E_{91}-E_{113}	9.04	0.74	1	6	10.8	2568	4
V_{93}-E_{110}	9.99	0.69	1	4	8.8	195	1
Y_{100}-V_{119}	10.2	0.71	1	6	9.2	68	1
L_{114}-L_{126}	8.58	0.57	0.75	2	6.8	1353	1
			1	6	2.8	401	1
Y_{115}-L_{126}	7.4	0.51	1	7	1	1	162
			0.5	5	3	54	1
K_{118}-E_{125}	4.76	0.45	0.75	3	1.8	584776	10
			1	1	3.8	19508	1
E_{120}-S_{142}	10.6	0.76	1	5	11.8	6272	1
			0.5	5	11.8	6235	3
E_{125}-E_{136}	17.0	0.71	1	5	3.8	18620	1
			0.5	1	7.8	$> 10^6$	4
L_{126}-L_{137}	18.2	0.64	1	3	5.8	$> 10^6$	40
			0.5	3	5.8	888	4
L_{126}-M_{148}	11.8	0.69	1	12	4.8	31383	5
Q_{127}-S_{142}	14.0	0.67	0.5	10	2	1	209
			1	7	5	266	1
Q_{127}-E_{147}	12.8	0.68	0.5	14	1.2	3754	2
			1	6	9.2	233	1
E_{128}-E_{147}	12.2	0.65	1	5	9.4	3849	2
E_{128}-M_{148}	11.4	0.65	0.5	14	1.2	1	35

			1	6	9.2	194	2
G_{129}-L_{137}	17.2	0.52	1	1	5.4	$> 10^6$	50
			0.5	2	4.4	4076	1
G_{129}-Q_{138}	13.4	0.51	1	2	5.2	26378	6
			0.5	7.2	-	3	-
H_{135}-A_{158}	11.9	0.68	0.33	2	15.6	15296	1
			1	8	9.6	748	3
E_{139}-H_{162}	13.5	0.73	0.5	4	13.6	202	1
			1	9	8.6	373	2
K_{140}-L_{159}	13.7	0.69	0.5	5	9.4	557	1
			1	4	10.4	1234	74
K_{140}-H_{162}	14.1	0.74	0.5	4	12.8	1044	2
			1	9	7.8	544	2
K_{140}-S_{167}	14.3	0.72	0.5	5	15	87	2
			1	9	11	440	2
S_{142}-A_{158}	11.7	0.73	1	3	9	107	2
M_{148}-A_{158}	17.1	0.58	0.33	7	1	1	90
			1	4	4	2140	1
M_{148}-Y_{166}	17.1	0.65	1	6	7.6	122	2
R_{149}-A_{164}	17.8	0.64	1	5	7	524	1
A_{158}-L_{170}	13.5	0.59	1	3	5.8	20059	17
A_{158}-R_{173}	13.4	0.63	0.75	7	4.2	4752	10
			1	4	7.2	3292	4
L_{159}-E_{169}	16.8	0.53	1	2	5.2	8155	1
L_{159}-L_{170}	13.4	0.52	1	1	7	16482	3
			1	6	2	1	60
R_{160}-L_{170}	11.8	0.47	1	4	3.2	44	1
T_{161}-G_{186}	11.9	0.67	1	9	10.2	2895	5
			0.5	16	3.2	1	53
L_{163}-Y_{192}	12.0	0.7	1	11	11.4	1472	4
			0.4	8	14.4	97	1
E_{169}-E_{179}	13.2	0.68	1	3	5	$> 10^6$	38
			0.5	6	2	14	1

L_{170}-L_{178}	14.1	0.56	1	2	4.4	188	4
R_{171}-L_{178}	11.4	0.47	1	2	3.6	309	1
			1	4	1.6	1854	1
			0.33	1	4.6	> 10^6	2
R_{171}-E_{179}	10.6	0.5	1	4	2.4	2398	2
			0.5	2	4.4	14324	1
R_{171}-A_{190}	13.2	0.66	1	10	5.2	365	1
E_{179}-L_{189}	15.4	0.56	1	7	1	3	710
			1	5	3	911	1
			0.75	2	6	73	3
L_{181}-L_{189}	21.2	0.57	1	4	2.4	1	30
L_{181}-E_{205}	19.3	0.71	1	4	15.2	2563	1
			0.5	13	6.2	1109	1
G_{185}-H_{199}	15.2	0.61	1	4	7.2	14206	7
L_{189}-T_{202}	18.4	0.62	1	3	7.4	39827	33
			0.75	3	7.4	66667	1
L_{189}-L_{203}	18.9	0.63	0.5	11.2	-	38	-
			1	2	9.2	1219	2
L_{189}-Q_{216}	13.2	0.7	1	10	10.8	443	2
A_{190}-T_{202}	20.9	0.65	1	2	7.6	937	6
			1	2	7.6	409	5
A_{190}-L_{203}	21.5	0.67	1	2	8.4	4875	6
			1	3	7.4	133	3
A_{190}-L_{211}	18.3	0.73	1	4	12	10702	1
T_{197}-L_{218}	13.1	0.68	1	9	7	190	1
S_{201}-L_{214}	8.62	0.6	1	1	8.6	12943	1
S_{201}-F_{225}	8.01	0.65	0.5	4	13.6	330	1
			1	8	9.6	183	1
L_{203}-E_{223}	7.15	0.62	1	9	5.4	119	1
S_{204}-L_{222}	7.31	0.61	1	11	1.8	87	1
E_{205}-F_{229}	8.23	0.65	1	9	8.6	1302	6
			0.5	4	13.6	7034	1
A_{207}-R_{215}	4.17	0.51	1	2	3.6	1056	1

P_{209}-S_{231}	7.61	0.64	1	12	4.8	110	1
			0.5	4	12.8	525	1
E_{212}-L_{219}	8.27	0.47	1	3	2.6	219	1
E_{212}-L_{222}	5.56	0.49	0.33	7.2	-	1	-
			1	6	1.2	73	2
D_{213}-L_{222}	6.13	0.49	1	4	2.4	138	1
L_{214}-L_{222}	5.12	0.4	1	4	1.6	142	2
R_{215}-L_{222}	3.47	0.35	1	3	1.8	8	2
P_{220}-T_{237}	8.6	0.68	1	6	7.6	131	1
E_{223}-E_{235}	10.1	0.56	1	4	5.6	5580	8
E_{223}-Q_{243}	12.2	0.67	1	7	9	162	1
S_{224}-Q_{243}	11.8	0.66	1	7	8.2	12238	3
F_{225}-F_{229}	6.44	0.36	1	1	2.2	222	1
F_{225}-L_{233}	11.0	0.49	1	4	2.4	158	1
L_{233}-T_{242}	10.6	0.63	1	6	1.2	1	10
L_{233}-Q_{243}	12.1	0.65	0.5	4	4	1711	7
			1	8	-	3	-
			0.5	3	5	18760	1
Y_{236}-Q_{243}	15.8	0.53	0.5	1	4.6	$> 10^6$	1
			0.5	2	3.6	$> 10^6$	21
			1	4	1.6	1	13

[a] Overlapping peptide

[b] HDX rate constant for the peptide in random coiled conformation. The unit of k_{rc} is min^{-1}.

[c] the power of the stretched exponential function used to fit theoretical and experimental D incorporation values.

[d] The number of exchangeable amide hydrogen atoms. Because the ratio between the protein solution and D_2O volumes is 1:4, then the actual number of exchangeable amide hydrogen atoms is 80% of the number of amide hydrogen atoms in the peptide. If the peptide exhibits biphasic HDX kinetics, then the total number of HDX sites is split in sites for each HDX phase.

[e] HDX protection factor ($Pf=k_{rc}/k_{hdx}$, k_{hdx} = HDX rate constant for the apoA-I folded peptide in spherical HDL; the unit of k_{hdx} is min^{-1}).

[f] For peptides that exhibit bimodal HDX kinetics each HDX mode is listed in a separate row .
